# pH-Responsive Modified Dextran Nanogel for Liver Targeted Doxorubicin Delivery

**DOI:** 10.3390/gels11100784

**Published:** 2025-10-01

**Authors:** Amin Raeisi, Mohammad Doroudian, Banafsheh Rastegari, Soliman Mohammadi-Samani, Abbas Behzad-Behbahani, Fatemeh Farjadian

**Affiliations:** 1 Pharmaceutical Sciences Research Center, School of Pharmacy, Shiraz University of Medical Sciences, Shiraz 7146864685, Iran; amin.raeisi.rx@gmail.com (A.R.); smsamani@sums.ac.ir (S.M.-S.); 2 Student Research Committee, Shiraz University of Medical Sciences, Shiraz 1433671348, Iran; 3 Department of Cell and Molecular Sciences, Faculty of Biological Sciences, Kharazmi University, Tehran 1571914911, Iran; doroudian@khu.ac.ir; 4 Diagnostic Laboratory Sciences and Technology Research Center, School of Paramedical Sciences, Shiraz University of Medical Sciences, Shiraz 7143914693, Iran; b.rastegari85@gmail.com (B.R.); behzadba@gmail.com (A.B.-B.); 5 Department of Pharmaceutics, School of Pharmacy, Shiraz University of Medical Sciences, Shiraz 7146864685, Iran

**Keywords:** smart nanogel, hydrogel, dextran, liver cancer, doxorubicin, drug delivery

## Abstract

A key obstacle to the efficacy of cancer drugs is the safe delivery of the drugs to the target site of the disease. Recent advances in nanomedicine have introduced smart hydrogel nanoparticles as promising, efficient, secure, and stimulus-responsive drug carriers. Herein, a bio-safe pH-sensitive nanohydrogel (NG) made of polyaminoethyl methacrylamide (AEMA)-grafted dextran was used as a carrier for liver-targeted doxorubicin (DOX) delivery. Lactobionate (SL) residue was conjugated to the prepared NG as a targeting agent, and DOX was also conjugated via Schiff base linkage. The synthesized structure was analyzed using ^1^HNMR, FT-IR, and size exclusion chromatography. DOX release was confirmed through UV-Vis spectroscopy. A pH-responsive manner in the DOX release profile was observed in a simulated medium with pH changes. In vitro toxicity assessment was performed in HepG2 and L929 cell lines, which have demonstrated the biosafety of the prepared hydrogel and its high effectiveness as an anticancer drug delivery system.

## 1. Introduction

Cancer is a significant contributor to global morbidity and mortality. It is expected that around 13.1 million people will die from cancer by the year 2030 [1]. Liver cancer is the sixth common cancer, with the third highest rate of fatality [2]. To address the impending crisis, considerable efforts have been devoted to developing potent anticancer agents, among which anthracyclines, particularly doxorubicin (DOX), stand out for their broad-spectrum activity, especially in hepatocellular carcinoma (HCC) treatment, a known type of LC [3]. However, the therapeutic utilization of DOX has been significantly curtailed due to its severe systemic toxicity, impacting the heart, kidney, brain, and liver [4]. In this way, a novel formulation of DOX based on nano-sized carrier encapsulation has been developed and listed as a nanopharmaceutical [5].

In recent decades, the importance of nanotechnology in direct drug delivery has grown, and progress has been made in controlling the location and speed of drug release [6,7]. Nanoparticles (NPs) of different types, such as metallic and non-metallic, have been engineered for drug, gene delivery, and precision medicine [8,9]. Compared to metallic NPs, non-metallic NPs have been shown to have several advantages: they are less toxic and more biocompatible, and they can be prepared through green synthetic methods [10]. Polymeric types of nanoparticles, such as micelles [11] and nanohydrogels, have gained recognition among other types [12]. Nanohydrogels, also known as nanogels, are three-dimensional networks of cross linked, hydrophilic synthetic or natural polymers. Nanogels are minute hydrogels ranging in size from 100 to 1000 nm. They boast three-dimensional polymer networks that are engineered through the chemical or physical cross-linking of polymer chains [13]. They can swell by absorbing water, but they do not open due to the presence of transverse connections between the polymer [14]. In the context of nanogels, these unique properties may include high surface area to volume ratio, enhanced permeability, increased reactivity, and the ability to respond to environmental stimuli. A wide range of applications exist for smart types of nanogels, which are capable of responding to chemical and physical stimuli. These gels have found significant use in the delivery of drugs, genes, and vaccines [12]. The properties of natural-based polymers, particularly polysaccharides, have led to their extensive research in fields such as tissue engineering, drug delivery, and other biomedical applications. These polymers are known for their biocompatibility and biodegradability, which make them highly desirable in these applications [15,16]. By placing sensitive groups in nanogel structures at various temperatures, pH levels, ionic strengths, and in light and electromagnetic fields, these stimulating systems can respond to environmental conditions.

Dextran is a biopolymer frequently referred to in drug and gene delivery due to offering benefits such as biodegradability, biocompatibility, ease of structural modification, and good interaction with hydrophilic agents [17]. Dextran has a glucose backbone with an α-1,6-glycosidic link. This structure can be easily modified by grafting polymer chains through radical polymerization, which makes it a suitable scaffold for medical applications [18]. Star-like polyacrylamide grafted dextran was prepared and utilized for pH-responsive DOX and cisplatin delivery in lung carcinoma [19]. In another effort, poly (vinylcaprolactone) grafted dextran was synthesized and encapsulated with vinblastine and assessed in breast cancer [20].

A key obstacle to the efficacy of cancer drugs is the effective delivery of drugs to cancer cells [21]. Today, using smart nanogel has offered innovative and new solutions to scientists in this field to design and use very effective, safe, and stimulus-responsive carriers to carry anticancer drugs [22]. Systems for delivering drugs have been designed mostly with pH responsiveness in mind, given that tumor tissues tend to be acidic [23].

Several pH-sensitive nanogel based drug delivery systems have been studied [24]. In previous studies conducted by Farjadian et al., pH responsive nanphydrogels were prepared via reversible addition fragmentation chain transfer polymerization and utilized for anticancer delivery purposes. Examples based on nanogel structure, drug, and type of cancer combating include poly(N-isopropylacrylamide-*co*-hydroxyethyl methacrylate-*co*-acrylamide) for DOX in colon cancer [25], histidine modified poly(aminoethyl methacrylate) for cisplatin delivery in colon [26], and poly(hydroxyethyl methacrylate) for methotrexate delivery in breast cancer [27].

The enhanced permeability and retention effect (EPR) is a passive targeting technique that allows NPs to accumulate in tumor tissue, increasing drug concentration significantly compared to free drugs, utilizing the pore size of tumor blood vessels [8,28]. Active targeting is a drug delivery technique where the ligand nanoparticle is specifically retained and absorbed by the targeted cells. Targeting ligands belong to the surface substances or the receptors that are overexpressed at particular sites [29]. A variety of receptors are significantly overexpressed on the surface of hepatoma cells in comparison to normal cells. These include the asialoglycoprotein receptor (ASGP-R), folate receptor (FR), epidermal growth factor receptor (EGFR), and transferrin receptor (TfR). Each receptor has a corresponding ligand—lactobionic acid, folic acid, 9B9 monoclonal antibody, and T7 peptide, respectively—that has been utilized to functionalize drug delivery systems and improve tumor cells’ ability to recognize and uptake nanoparticles [30,31]. A transmembrane protein called the receptor (ASGP-R) connects the cell surface to membrane-bound intracellular compartments. It is recycled after binding galactose-terminal molecules and transferring them to the cell’s lysosomes for catabolism [32]. In Wu et al.’s study, for the colon-targeting release of DOX, a targeting CS/SALG hydrogel was developed by double cross-linking chitosan (CS) and sodium alginate (SALG). According to in vivo and in vitro assessments, the DOX-loaded structure demonstrated clear inhibition of tumor cells. These findings indicated oral administration of this formulation for DOX transport that targets the colon [33].

Herein, a pH-sensitive nanogel (NG) loaded with the anticancer drug DOX was prepared. The NG synthesis was conducted on dextran polymer, which was modified by the grafting technique with poly(aminoethyl methacrylate) (PAEMA) and cross-linked with poly(ethylene glycol) dimethacrylate (PEG-DMA). In the next step, DOX was conjugated to the amine group of the PAEMA polymer through a Schiff base bond to create a pH-responsive anticancer delivery system. The final product was modified with lactabionate (SL) residue as a targeting agent of the HCC cells receptor. The product of each step was characterized using various techniques, and the pH responsive drug release profile of the DOX-NG has been proven. Finally, the cytotoxicity effect of the prepared materials was assessed in vitro using the HepG2 and L929 cell lines.

## 2. Results and Discussion

### 2.1. Synthesis and Characterization of the Polymers

**Figure 1:**
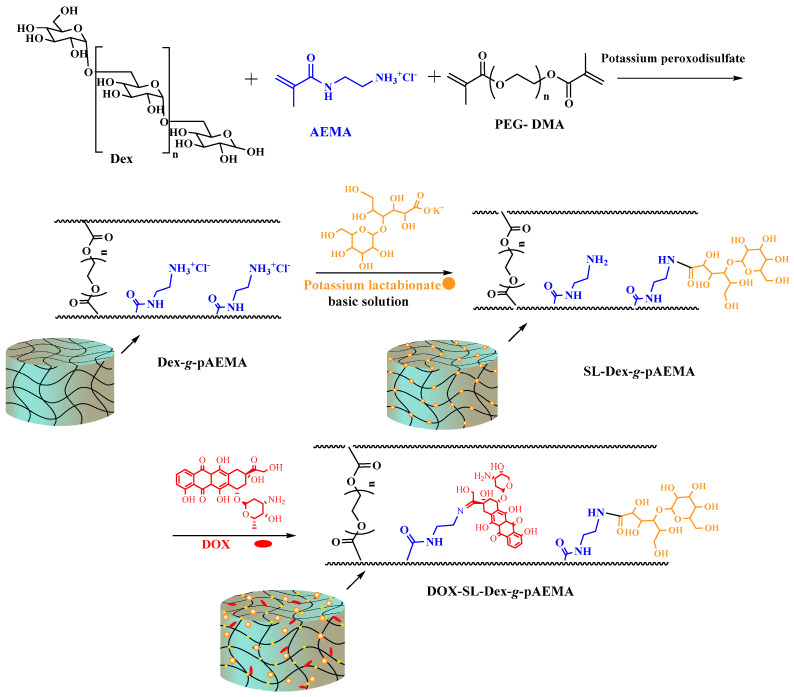
A schematic synthesis of Dex-SL-Dex-*g*-pAEMA. In the first step, pAEMA (blue) was grafted to Dex via free radical polymerization in the presence of potassium peroxodisulfate. In the second step, potassium lactabionate (orange) was reacted with Dex-*g*-pAEMA through carbodiimide chemistry. In this process, lactobionate was activated by EDC and NHS and reacted with Dex-g-pAEMA in a basic solution. In the third step, DOX (red) was reacted with SL-Dex-g-pAEMA, which resulted in DOX conjugation via Schiff base reaction.

The implementation of a synthetic approach led to the synthesis of DOX-Dex-*g*-pAEMA, and it is used for conjugating DOX and SL as a targeting ligand, as is depicted in Figure 1.

The synthesis process was performed using various initiators, such as azobisisobutyronitrile and benzoyl peroxide, but the polymerization processes did not proceed successfully. Finally, potassium peroxodisulfate, as a free radical-forming initiator, demonstrated the best results for polymerization.

**Figure 2:**
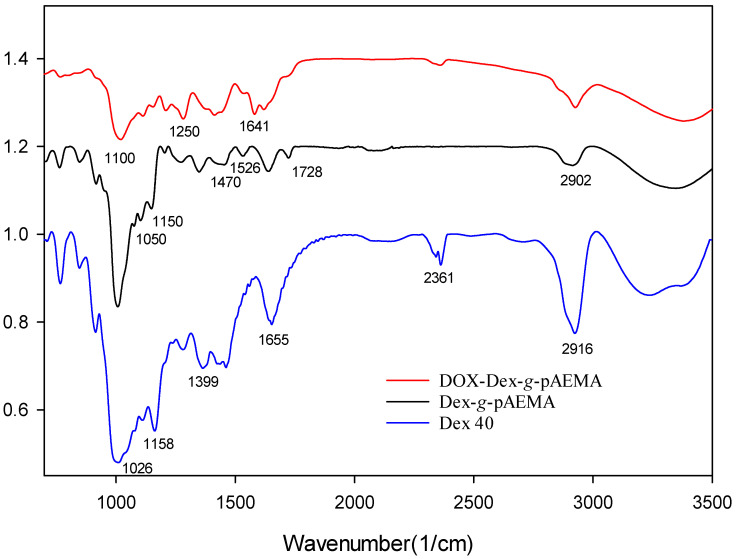
FT-IR spectra of Dex 40 (blue), Dex-g-pAEMA (black), and DOX-Dex-g-pAEMA (red).

The successful synthesis of NGs was proved through ^1^HNMR and FT-IR analysis. The peaks observed in the FT-IR spectrum indicate the presence of characteristic functional groups in the structure of NGs when compared with Dex-40 (Figure 2). The Dex-*g*-pAEMA spectra show that the C=O stretch band appeared at 1637 cm^−1^. Additionally, the formation of amide groups in Dex-*g*-pAEMA resulted in the appearance of peaks around 1470 cm^−1^ and the shoulder at 1526 cm^−1^. In the FT-IR spectrum of DOX-Dex-*g*-pAEMA, the peak at 1250 cm^−1^ is related to the C=N band of the Schiff base, and the peak at 1641–1660 cm^−1^ represents the characteristic bands of C=C groups related to aromatic rings of DOX.

**Figure 3:**
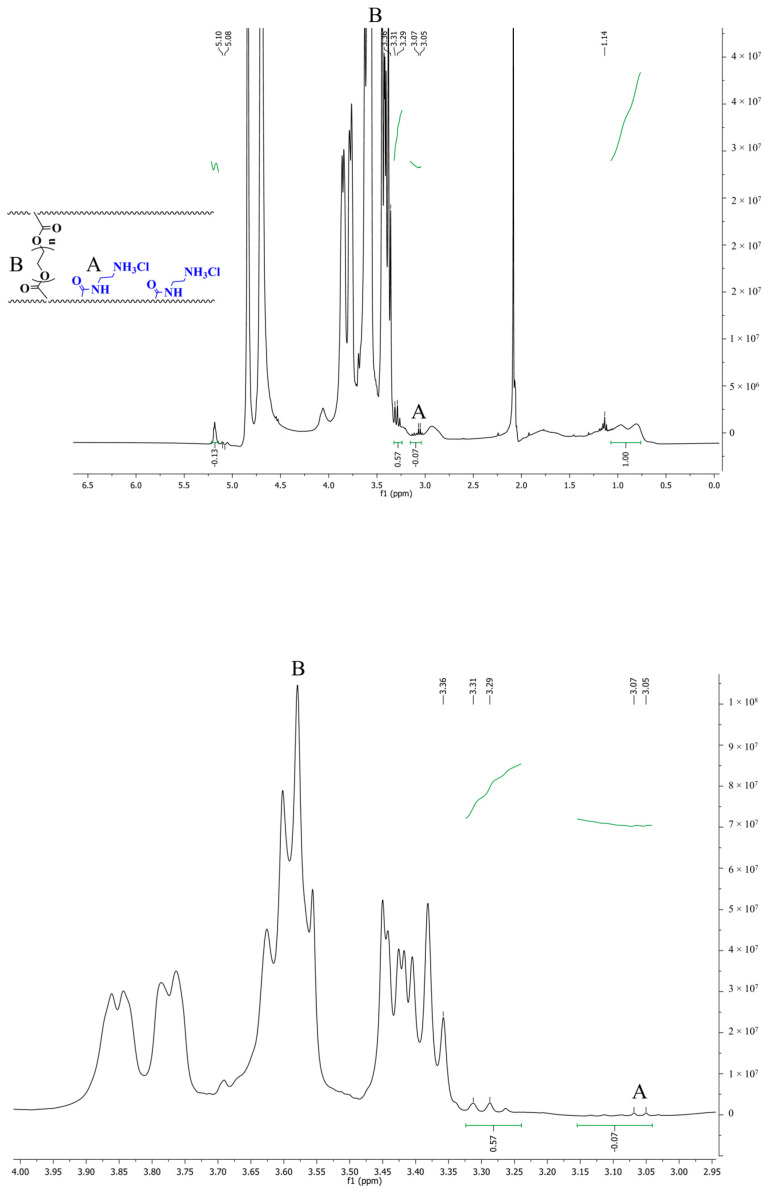
^1^HNMR spectrum of Dex-*g*-pAEMA in D_2_O (top image is full spectrum and Bottom image is magnified spectrum) (Letters A and B correspond to-CH_2_- band of AEMA and PEG-DMA as depicted in chemical formula in the Figure 3, respectively).

Furthermore, the ^1^HNMR spectrum validated the FT-IR findings (Figure 3). ^1^HNMR spectra of Dex-*g*-pAEMA show that the peak at ~3 ppm is associated with CH_2_ of AEMA, and the methylene groups of PEG-DMA are represented by peaks at ~3.5 ppm. The sharp peak at 4.9 ppm is associated with D_2_O’s chemical shift. Based on the area under the peak in the magnified ^1^HNMR images and the index peaks of dextran and pAEMA, it was determined that 21% of the NGs’ weight belongs to pAEMA and 1.5% to the crosslinker.

### 2.2. Gel Permeation Chromatography (GPC)

SEC was conducted to evaluate the MW of Dex-*g*-pAEMA. Dextran 40 kDa and dextran 70 kDa were used as standards. The polymer’s weight was estimated at 47 kDa. These results indicate that 18% of pAEMA is present in the NG’s structure, which is close to the data obtained from ^1^HNMR.

### 2.3. Morphology and Size Assessment

**Figure 4:**
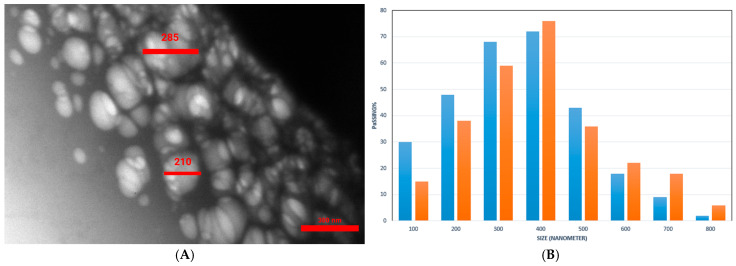
(**A**) TEM image (left) and (**B**) DLS histogram of Dex-*g*-pAEMA (blue) and DOX-Dex-*g*-pAEMA(orange) (right).

TEM imaging and DLS were employed to determine the morphology and size of Dex-*g*-pAEMA and DOX-Dex-*g*-pAEMA. According to TEM (Figure 4A), the Dex-*g*-pAEMA particle sizes ranged between 187 and 310 nm. The TEM analysis of the sample reveals that Dex-*g*-pAEMA consists of small spherical particles. Moreover, the DLS histogram showed that both particles have hydrodynamic diameters between 100 and 800 nm, with the frequency of the particle size being less than 400 nm (Figure 4B). The poly dispersity index (PDI) of Dex-*g*-PAEMA was determined to be 1.57.

Zeta potential measurement makes it possible to predict the storage stability of NHs, since charged particles aggregate less frequently [34]. According to the results of zeta potential measurements, Dex-*g*-pAEMA has a charge of +18 mV, while this dropped to −10.5 mV in the DOX-conjugated formula. This observation arises from the positive nature of pAEMA. The sharp drop results from the neutralization of the structure during DOX conjugation.

### 2.4. DOX Conjugation and Release Pattern

**Figure 5:**
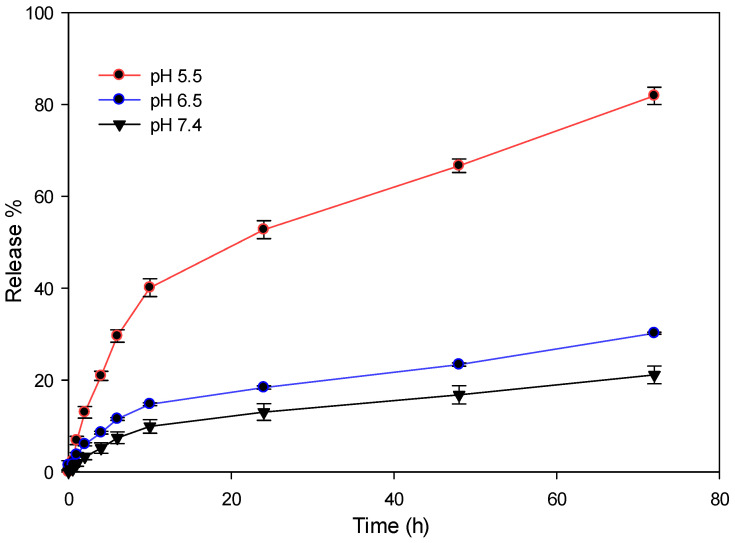
Drug release pattern from DOX-Dex-g-pAEMA at various time intervals (0, 0.5, 1, 2, 4, 6, 12, 24, 48, and 72 h) in pH 5.5, 6.5, and 7.4.

Using the Schiff base reaction, DOX was conjugated to the amine group of the Dex-*g*-pAEMA structure. The amount of DOX was determined by the desorption technique through UV-Vis spectroscopy to be 10% of the total weight of DOX-Dex-*g*-pAEMA. The Schiff base bond would provide a pH-sensitive delivery system. Cancer cells exhibit lower pH levels in comparison to non-malignant cells, which can enable this system to release DOX into the acidic environment of tumor cells [35]. Figure 5 displays the release pattern of DOX at three distinct pH levels: 5.5, 6.5, and 7.4. The minimum number of releases occurred at a pH value of 7.4 in a period of 72 h, to be 21%. A decrease in pH to 6.5 results in an increment in drug release of approximately 30% in 72 h. The highest release percentages (81%) were observed over 72 h at a pH of 5.5, which indicates the pH-responsive properties of DOX-Dex-*g*-pAEMA.

### 2.5. In Vitro Toxicity Evaluation

**Figure 6:**
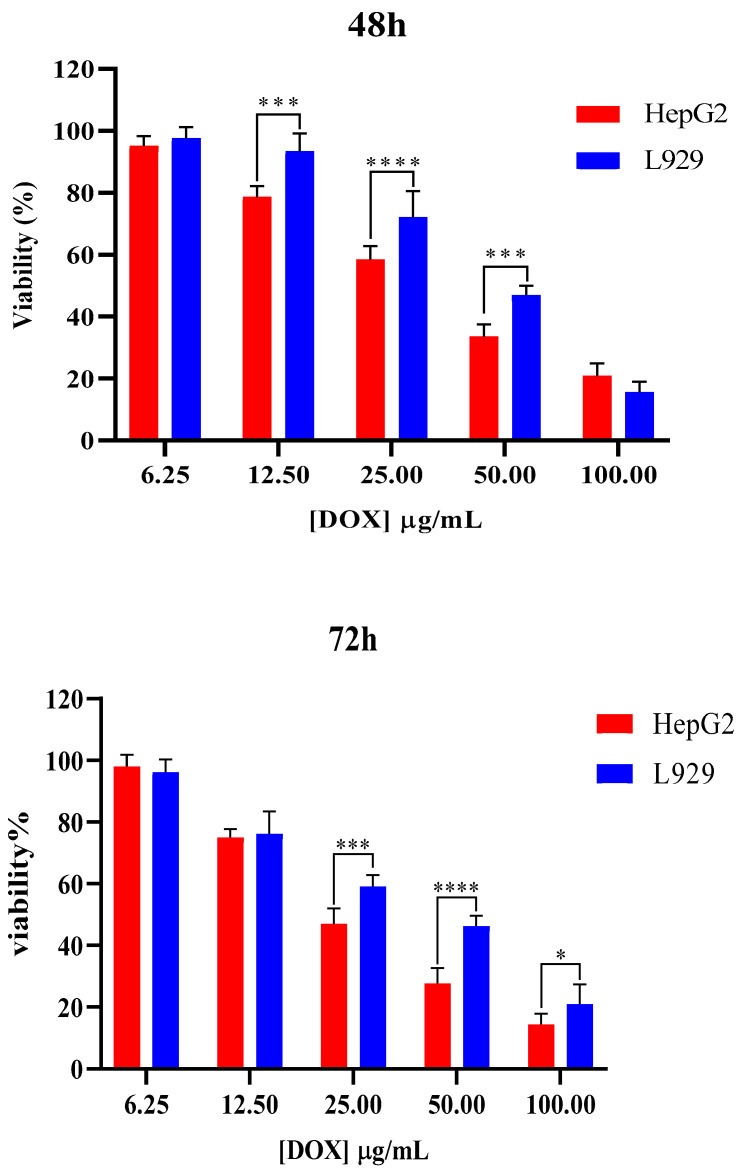
Cell viability assessments regarding different concentrations of free DOX in HepG2 and L929 cell lines. Significance levels were categorized as follows: * for *p* ≤ 0.05, *** for *p* ≤ 0.001, and **** for *p* ≤ 0.0001.

**Figure 7:**
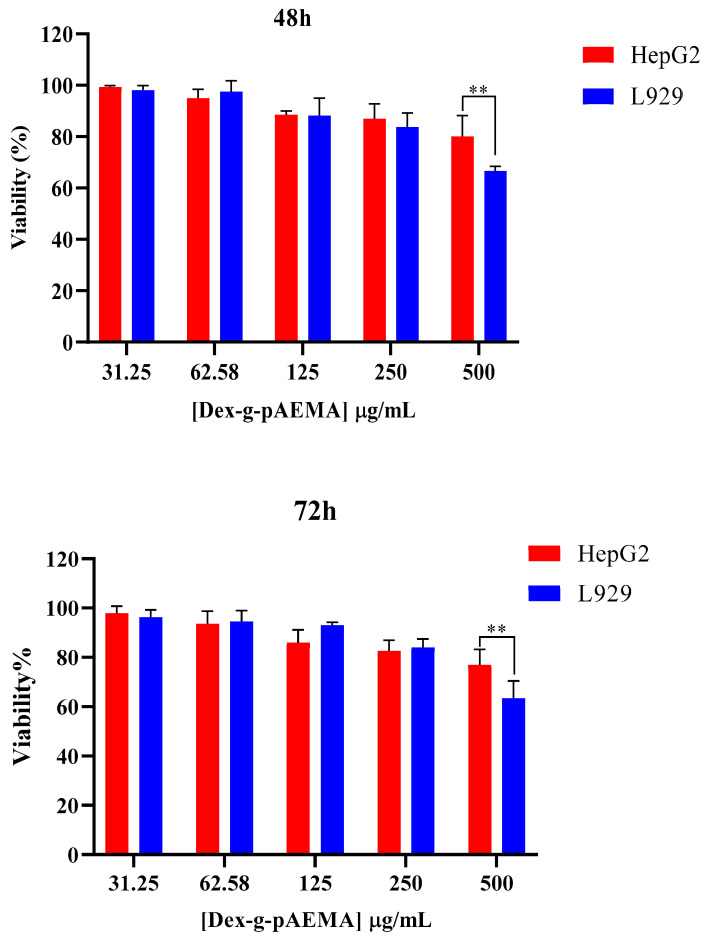
Cell Viability assessment of HepG2 and L929 cell lines in the presence of Dex-g-pAEMA. Significance levels were categorized as follows: ** for *p* ≤ 0.010.

**Figure 8:**
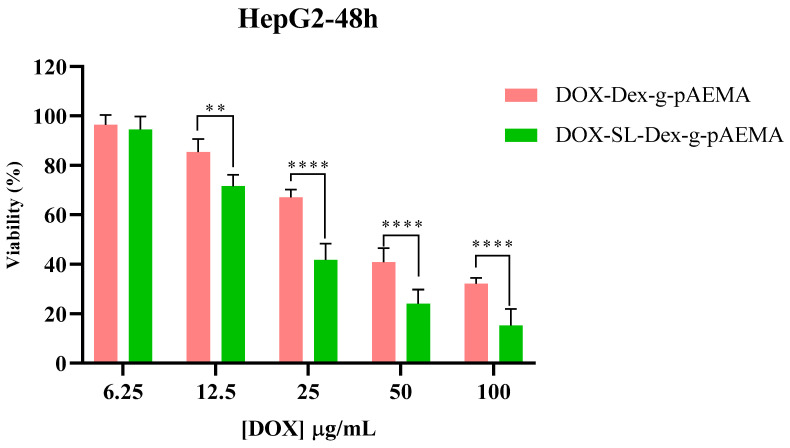

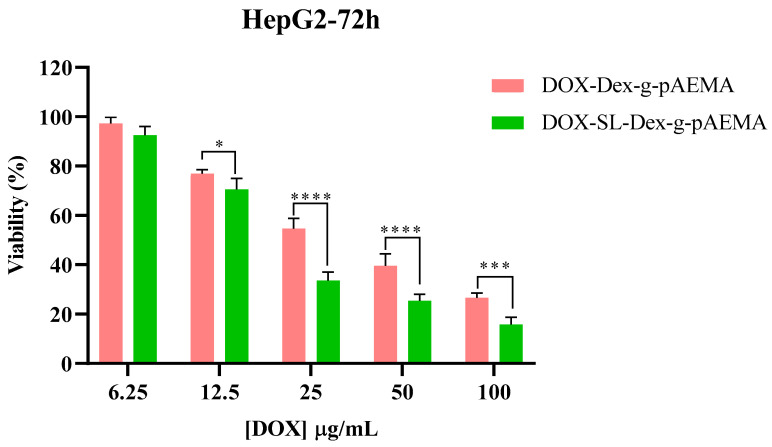
Cell viability assessment of the DOX-Dex-g- and DOX-SL-Dex-g-pAEMA in HepG2 cell line. Significance levels were categorized as follows: * for *p* ≤ 0.05, ** for *p* ≤ 0.01, *** for *p* ≤ 0.001, and **** for *p* ≤ 0.0001.

**Figure 9:**
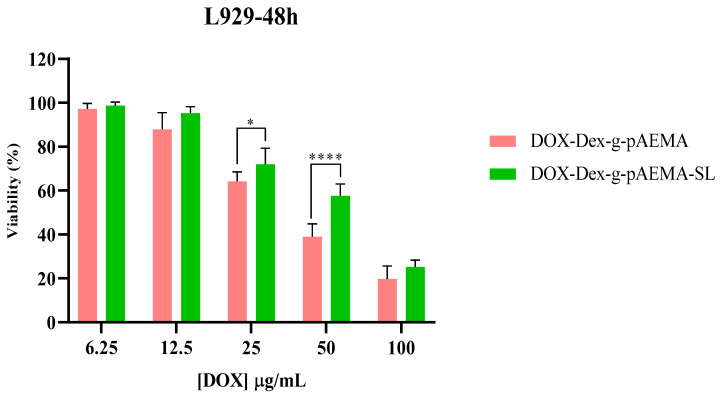

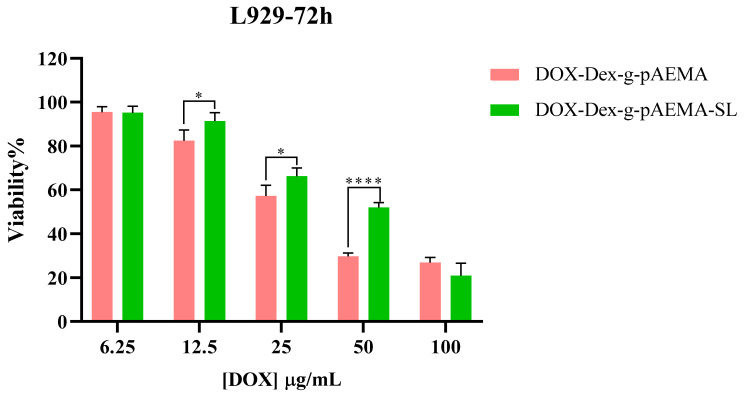
Cell viability assessment of DOX-Dex-g-pAEMA and DOX-SL-Dex-g-pAEMA in the L929 cell line. Significance levels were categorized as follows: * for *p* ≤ 0.05 and **** for *p* ≤ 0.0001.

The cytotoxicity of NGs and free DOX was assessed using the MTT assay on HepG2 and L929 cell lines, and the results are presented in Figure 6, Figure 7, Figure 8 and Figure 9. The toxicity of different concentrations of free DOX, Dex-*g*-pAEMA, DOX-Dex-*g*-pAEMA, and DOX-SL-Dex-*g*-pAEMA was evaluated for over 48 and 72 h.

The anticancer potential and biocompatibility of the DOX, Dex-*g*-pAEMA, and DOX-Dex-g-pAEMA were assessed using the MTT colorimetric assay following 48 and 72 h of treatment across HepG2 liver cancer cell lines (See Figure 6, Figure 7, Figure 8 and Figure 9). As illustrated in Figure 6, DOX demonstrated significant cytotoxic activity against liver cell lines, while also being relatively more biocompatible against L929 normal fibroblasts up to 25 and 12.5 µg/mL within 48 and 72 h post-incubation, respectively. On the other hand, the trend of diminished cell viability in treated HePG2 and L929 cells was significant (*p* = 0.0083; *p* = 0.0076). Additionally, the changes in cytotoxicity between HePG2 and L929 cells at 48 h at concentrations of 12.5, 25, and 50 micromolar indicate the selectivity of DOX between cancerous and normal cells. This trend was also observed at 72 h of incubation at concentrations of 25, 50, and 100 µg/mL.

Dex-*g*-pAEMA showed remarkable cell biocompatibility up to 250 µg/mL against both cancerous and normal cells, even after 72 h of incubation (See Figure 7). The results showed that L929 cells only exhibited toxicity at a concentration of 500 µg/mL of the polymer, and a significant difference between L929 and the HePG2 cell line was observed at both the 48- and 72 h time points (*p* = 0.004; *p* = 0.009).

After 48 and 72 h of incubation, DOX-SL-Dex-*g*-pAEMA induced toxicity more efficiently than DOX-Dex-*g*-pAEMA in HepG2 cells (See Figure 8). Cytotoxicity of the DOX-Dex-g-pAEMA and DOX-SL-Dex-*g*-pAEMA on the HePG2 cell line demonstrated that the presence of SL significantly increases the cytotoxicity of the drug delivery system within the concentration range of 12.5 to 100 µg/mL of DOX at both 48 and 72 h. In contrast, the cytotoxicity of the polymer in the presence of SL, compared to the sample without SL, is significantly evident, especially at DOX concentrations of 25 and 50 µg/mL, as shown in Figure 9. Also, HepG2 cells are more susceptible to DOX-SL-Dex-*g*-pAEMA, especially after 72 h of incubation. In contrast, DOX-SL-Dex-*g*-pAEMA showed remarkable biocompatibility compared to Dex-g-pAEMA even after 72 h of incubation (See Figure 9). These differences are best described using the half maximal inhibitory concentration (IC50).

As seen in Table 1, DOX-SL-Dex-*g*-pAEMA shows higher cytotoxicity compared to free DOX and DOX-Dex-*g*-pAEMA in the HepG2 cell line (Table 1). However, Table 1 indicates that the L929 cell line’s viability in DOX-SL-Dex-g-pAEMA is higher than that of DOX-Dex-*g*-pAEMA. As a result, lactobionate, a ligand with a galactose residue, targets the hepatocyte membrane’s highly concentrated asialoglycoprotein receptor (ASGP-R), which is healthier for normal cells and more cytotoxic to cancerous cells (Figure 9).

The significant differences in cytotoxicity between the normal (L929) and the HCC (HepG2) cell lines indicate notable selectivity of both DOX -Dex-*g*-pAEMA and DOX-SL-Dex-*g*-pAEMA compared with free DOX. The Selectivity Index (SI) is a critical metric in anticancer drug development, quantifying a compound’s specificity for cancerous cells over normal cells. It is calculated as the ratio of a drug’s cytotoxicity to normal cells (IC_50_ normal) versus cancerous cells (IC_50_ cancer). A higher SI indicates greater selectivity, meaning the drug effectively targets cancer cells while sparing healthy ones. While the 1 < SI < 2 is considered selective, the 2 < SI < 10 is often recommended for further investigation as a potential therapeutic [36]. The Selective Index (SI) values reported in Table 1 showed that DOX-SL-Dex-*g*-pAEMA has the highest SI values (greater than one), suggesting its enhanced safety for use. Compared to free Doxorubicin, which has SI values of 1.28 < SI < 1.99, DOX-Dex-*g*-pAEMA formulations demonstrated lower potency for inhibiting the proliferation of the tested cancer cell line than DOX-SL-Dex-*g*-pAEMA with SI values of 2.40 < SI < 2.64. Overall, the DOX-SL-Dex-*g*-pAEMA holds promise as a therapeutic candidate for HCC.

### 2.6. Cellular Uptake Study

**Figure 10:**
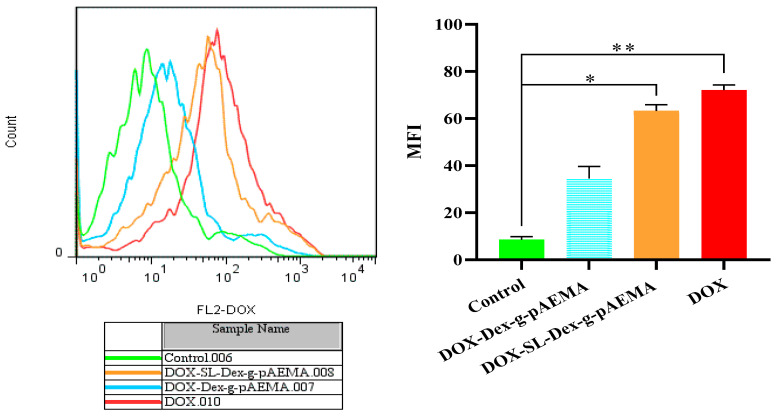
The cellular uptake of DOX, DOX-Dex-*g*-pAEMA, and DOX-SL-Dex-*g*-pAEMA against the HepG2 cell line within 6 h was compared with untreated control. Significance levels were categorized as follows: * for *p* ≤ 0.05, ** for *p* ≤ 0.01.

The cellular uptake study revealed successful uptake of DOX-Dex-g-pAEMA and DOX-SL-Dex-g-pAEMA compared with free DOX. As illustrated in Figure 10, the mean fluorescence intensity of red fluorescence drug, DOX, was significantly elevated after exposure to DOX-SL-Dex-g-pAEMA and free DOX, respectively (*p* = 0.031 and *p* = 0.0077).

### 2.7. Discussion

In this research, a smart NG was developed for DOX delivery. In the first step, dextran was modified by pAEMA and crosslinked with PEG-DMA via the free radical polymerization technique. Then, SL as a targeting agent and DOX as an anticancer agent were conjugated to the modified structure. The structure was characterized by the ^1^HNMR and FT-IR. Then, the size of the particle was assessed using DLS and TEM techniques, which have proved well formation of the nanogel. According to the release pattern of DOX from DOX-Dex-*g*-pAEMA, in acidic environments, this NG can respond to pH changes and release DOX efficiently at acidic pH (5.5).

Research has shown that a 40–400 nm nanoparticle size range is appropriate to ensure extended circulation and increased accumulation in the tumor with the EPR, with decreased renal clearance [37]. The hepatocyte membrane cell line known as HepG2 expresses the asialoglycoprotein receptor (ASGP-R). Wang et al. synthesized a novel galactosylated lipid, (5-Cholesten-3b-yl) 4-oxo-4-[2(lactobionyl amido) ethylamido] butanoate (CHS-ED-LA) and incorporated it into liposomes to enhance the liver-targeting efficiency of DOX and utilize lactobionic acid as a targeting ligand to ASGP-R hepatocyte cells. This modification increased hepatic targeting efficiency to 64.6%, compared to 21.8% for conventional liposomal DOX. The presence of lactobionic acid enhanced the specificity of drug delivery systems that target hepatocytes by facilitating receptor-mediated uptake via the ASGP-R [38]. The capacity of lactobionate (SL) to bind to asialoglycoprotein (ASGPR) receptors found in hepatic cells makes it suitable for use in drug delivery systems [39]. According to the IC_50_ values, the MTT assay results showed that the viability of the L929 cell line in DOX-SL-Dex-g-pAEMA is less than that of DOX-Dex-g-pAEMA. Consequently, lactobionate, a ligand that targets the ASGP-R receptor on the hepatocyte membrane, may cause DOX-SL-Dex-*g*-pAEMA to have receptor-mediated endocytosis in HepG2 while having a lower cell entrance on normal cell lines. This targeted strategy makes DOX-SL-Dex-*g*-pAEMA a toxic agent for HCC cells while keeping it safe to normal cells.

The key issue underlying the effectiveness of antitumor agents is considered to be the sensitivity of tumor cells to them, and not their toxicity. Due to the massive and often mosaic mutation of tumor cells within one solid neoplasm, the selection of an antitumor therapy regimen is carried out using data on the type of cancer cells and the mechanisms underlying their sensitivity to cytostatics [40]. In this regard, herein, the relative sensitivity of the delivery systems is mostly related to the smart tissue penetrations of the nanoplatforms and efficient drug payloads on tissue microenvironments and/or intra cellular spaces like cytoplasm or lysosome. Key to addressing tumor sensitivity, our nanogel exhibits pH-responsive drug release behavior. The release studies showed that DOX is efficiently released from the nanogel in acidic environments (pH 5.5), which closely mimic the tumor microenvironment. This pH-triggered release mechanism ensures that DOX is preferentially delivered and released within the acidic milieu of tumor tissues, thereby increasing the local concentration of the drug at the site of sensitive tumor cells while minimizing exposure to normal tissues. By incorporating a targeting ligand (SL), our system further enhances the specificity of drug delivery to tumor cells, potentially overcoming the issue of heterogeneous cell populations within a single tumor. This targeted, stimuli-responsive approach is designed to maximize the cytotoxic effect on sensitive tumor cells, which is in line with the current paradigm in oncology that prioritizes tumor cell sensitivity over nonspecific toxicity.

## 3. Conclusions

A bio-safe, pH-responsive NG was developed for liver-targeted DOX delivery. Lactobionate was used for targeting and DOX was used as the chemotherapeutic agent. Dextran backbone was modified with a successful free radical polymerization technique using dextran, AEMA, and PEG-DMA. ^1^HNMR and FT-IR data show that the structure of dextran was successfully modified. DLS and TEM confirm the formation of nanogel. The findings from the drug release experiments conducted at various pH levels (5.5, 6.5, and 7.4) indicate a pH responsive release pattern, with approximately 81% of the drug being released at a pH of 5.5. In vitro cellular studies have indicated that the NG structure is biocompatible and demonstrates significant cytotoxicity toward hepatocyte cells due to lactobionate acting as a ligand that targets the ASGP-R receptor. DOX-SL-Dex-*g*-pAEMA is more effective than free DOX and holds promise as an anticancer agent. Overall, NGs demonstrate significant potential as drug delivery systems in cancer treatment.

## 4. Materials and Methods

### 4.1. Materials and Instruments

Doxorubicin hydrochloride (DOX-HCl) was supplied by Saba (Istanbul, Turkey). Dextran 40 and Dextran 70 KD, poly(ethylene glycol) dimethacrylate (PEG-DMA), 1-ethyl-3-(3-dimethylaminopropyl) carbodiimide hydrochloride/(EDC), and N-hydroxysuccinimide (NHS) were prepared from Sigma-Aldrich (Darmstadt, Germany). Potassium lactobionate was purchased from Henan Tianfu Chemical Co. (Zhengzhou, China). Aminoethyl methacrylamide (AEMA) was provided by PolyScience (Niles, IL, USA). Ethanol was obtained from Kimia Mavad (Najafabad, Iran). HepG2 and L929 cell lines were acquired from the Pasteur Cell Bank (Tehran, Iran). High glucose DMEM and RPMI1640 media, penicillin–streptomycin, fetal bovine serum (FBS), phosphate-buffered saline (PBS), and L-glutamine were supplied by Shellmax (Taizhou, China).

In this investigation, proton nuclear magnetic resonance (^1^HNMR) (Bruker 400 MHz spectroscopy (Rheinstetten, Germany)) was performed using D_2_O as the solvent. Fourier-transform infrared spectroscopy (FT-IR) spectra were measured with Vertex 70 (Selb, Germany) using KBr powder as a control to characterize the structures at different stages of synthesis in the range of 400–4000 cm^−1^. The hydrodynamic diameter of polymers was determined using dynamic light scattering (DLS) (NANOflex), and the zeta potential was determined by a zeta sizer (Microtrac (York, PA, USA)). The sample was prepared by using deionized water as the solvent to prepare three 3 mL samples of Dex-*g*-pAEMA polymer at a concentration of 5 mg/mL. To achieve a homogeneous dispersion, each sample underwent probe sonication (Hielscher Company, Teltow, Germany, Germany) for three minutes. To measure the UV spectra of the DOX-loaded sample, ultraviolet-visible (UV-Vis) spectroscopy CECIL instrument CE7250 (Cambridge, UK) was used. Transmission electron microscopy (TEM) was carried out with Philips CM 10–100 kV (Eindhoven, The Netherlands). To prepare the sample, grids were formed by dropping 500 µg/mL of Dex-*g*-pAEMA into a solution. After taking pictures at room temperature, the grid was incubated at 42 °C, and then more images were taken. For UV-Vis records, a Biotrak ELISA plate reader (Amersham Pharmacia Biosciences, Piscataway, NJ, USA) was used. The 32 mm wide dialysis tubing from Sigma (St. Louis, MO, USA) has a MWCO of 12,400. The molecular weight (MW) of NG was analyzed using size exclusion chromatography (SEC) (iZON Science, Oxford, UK). Dextran 40 kDa and dextran 70 kDa were used as standards. The Azura Knauer HPLC system with an RI detector (Berlin, Germany) was utilized in this analysis, with the PSS SUPREMA, which had a particle size of 10 µm (300 mm × 10,000 Å) as the column.

### 4.2. Synthesis of NG

#### 4.2.1. Synthesis of Dextran-Grafted Poly(Aminoethyl) Methacrylate (Dex-g-pAEMA)

Dextran (40 kDa) was grafted with pAEMA and cross-linked with PEG-DMA (Mn:550) by free radical polymerization. For this purpose, the mixture of ethanol/water (36/24 cc) in a three-neck flask was degassed with N_2_ atmosphere for 20 min. Then, dextran (0.6 g~3.73 mmol anhydrous glucose repeating unit) and potassium peroxodisulfate (25 mg~0.0925 mmol) were added to the solutions and mixed at room temperature for an hour (h). After that, PEG-DMA (25 µL~27.75 mg~0.05 mmol) and AEMA (250 mg~164.63 mmol) were added to the mixture (the optimized molar percent ratio of peroxodisulfate–PEG-DMA–AEMA regarding glucose mmole of dextran was 2.4:1.3:41.31). A balloon filled with nitrogen was connected to one neck of the flask, while a condenser was attached to another neck, and the third neck of the balloon was capped. After sealing the flask, it was stirred in an oil bath at 80 °C for 48 h. Finally, dialyzing and lyophilizing were used to purify Dex-*g*-pAEMA.

#### 4.2.2. Conjugation of DOX (DOX-Dex-g-pAEMA)

By using the previously reported procedure [41], a Schiff base reaction resulted in the conjugation of DOX. In summary, Dex-*g*-pAEMA (80 mg) and triethylamine (Et_3_N) (20 µL) were reacted in deionized water (DIW) (12 cc). The mixture was subjected to stirring (500 rpm) at ambient temperature for 1 h. Then, DOX (10 mg) was incorporated into the mixture and left to stir (at 500 rpm) at room temperature in a dark place for 48 h. Subsequently, to eliminate free DOX, the mixture underwent dialysis in DIW for 6 h and was freeze-dried.

##### Preparation of DOX Calibration Standard Curve

A series of doxorubicin standards was prepared in DIW at concentrations of 2.5, 5, 10, 15, 20, and 100 µg/mL, with three replicate samples for each concentration. UV-Vis spectrophotometry was used to measure each solution’s absorbance at a wavelength of 232 nm. After that, a calibration curve for doxorubicin in aqueous media was created.

**Figure 11:**
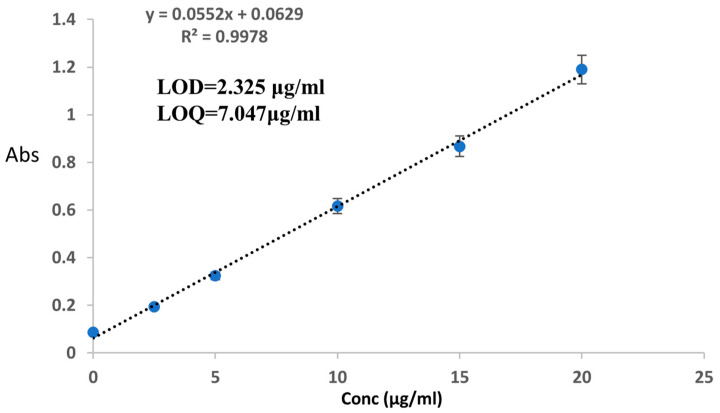
Calibration curve of DOX for validation.

The calibration curve for DOX prepared in DIW exhibited a linear relationship within the concentration range studied. As depicted in Figure 11, the calibration curve demonstrated a high degree of linearity with a regression coefficient (R^2^) of 0.9978 and a slope of 0.0552.

#### 4.2.3. Conjugation of Potassium Lactobionate (SL-Dex-g-pAEMA)

Potassium lactobionate (105 mg) was initially added to the flask containing DIW (10 mL). After that, the EDC (0.25 mmole) and NHS (0.4 mmole) were added to the mixture and stirred at 500 rpm at ambient temperature for one h. In the next step, triethylamine (Et_3_N) (80 µL) and Dex-*g*-pAEMA (240 mg) were added. The flask was tightly capped and placed within an oil bath and stirred at 50 °C for 48 h. Finally, the solution was dialyzed in a dialysis bag in 1 L of DIW and then lyophilized for 24 h.

#### 4.2.4. Conjugation of DOX (DOX-SL-Dex-g-pAEMA)

DOX conjugation to SL-Dex-*g*-pAEMA was performed according to the previous method explained in Section 4.2.2.

### 4.3. Release Pattern of DOX

Dialysis was used to determine the release profile of DOX from DOX-Dex-*g*-pAEMA against PBS solutions with pH values of 5.5, 6.5, and 7.4, respectively. Briefly, a dialysis bag (molecular cut-off: 10 kDa) was filled with 10 mL of DOX-Dex-*g*-pAEMA (1 mg/mL) suspension, and the bag was placed in 100 mL of the buffer solution release medium at 37 °C while being shaken lightly. At each time interval (0.5, 1, 2, 4, 6, 10, 24, 48, 72 h), 2 mL of the release medium was gathered and replaced with an equivalent volume of fresh medium. The DOX release amount was determined by UV-Vis spectrophotometry at 232 nm. The cumulative release percentage of DOX was calculated by the following equation (Equation (1)).

(1) $$\mathrm{Cumulative}\mathrm{release}\mathrm{percent}\mathrm{of}\mathrm{DOX}=\frac{V_{e}\;\sum_{i=1}^{n-1}C_{i\;}+V_{0\;\;}C_{0}}{m_{0}}\times 100\%$$

### 4.4. In Vitro Cellular Studies

Toxicity MTT assay was conducted on HepG2 (human liver cancer) and L929 normal cell lines. The HepG2 and L929 cells were grown in high-glucose DMEM and RPMI1640 medium with 10% FBS supplemented with 1% (*V*/*V*) penicillin–streptomycin in a humidified medium with 5% CO_2_, respectively. Cell survival was investigated by MTT assay. In summary, 5.0 × 10^4^ MCF-7 cells were pre-cultured in each well in a 96-well plate, and after 16 h in the incubator, different concentrations of Dex-*g*-pAEMA (31.25, 62.5, 125, 250, and 500 µg/mL), DOX (6.25, 12.5, 25, 50, and 100 µg/mL), DOX-Dex-*g*-pAEMA, and DOX-SL-Dex-*g*-pAEMA (equal 6.25, 12.5, 25, 50, and 100 µg/mL content of DOX) were added to the cells in a fresh media within 48 and 72 h. Finally, a fresh medium of MTT solution with a final concentration of 0.50 mg/mL was added to each well and incubated for 4 h. Lastly, the containing solution of culture medium was removed, and DMSO (100 µL of 100% purity) was added to dissolve the crystalline formazan. Then, the BMG Spectro Nano Elizabeth Reader read absorbance of the samples at two wavelengths (λ: 570 and 630 nm). To calculate viability percent, the following Equation was used:

%Cell viability = [A_T (sample)_/A_T (control)_] × 100 (2)

where the AT is A_570_–A_630_.

Data were gathered from triplicate independent experiments and represented as mean ± standard deviations. The statistical significance of results evaluated using non-parametric equivalent of two-way ANOVA is Friedman’s test.

### 4.5. Cellular Uptake Study

The total population of 2 × 10^5^ HepG2 cells was pre-cultured overnight. Then, cells were exposed to DOX (40 µM) and DOX-Dex-g-pAEMA or DOX-SL-Dex-*g*-pAEMA (equal to 40 µM) for 6 h. Then, cells were trypsinized and the Auto-Red fluorescence emission of the DOX was monitored with a 488 nm excitation wavelength and a 575 nm detection channel (FL2) using BD FACS Calibur^TM^ flow cytometry (BD Biosciences, San Jose, CA, USA). The statistical evaluation of Mean Fluorescence Intensity (MFI) was examined using a Student’s *t*-test of three independent experiments.

## Figures and Tables

**Table 1:** IC_50_ values (ppm) of samples in HepG2 and L929 cell lines after 48 and 72 h.

Cell Line	HepG2		L929	
Sample (µg/mL)	48	72	48	72
DOX	39.27 ± 2.21	24.64 ± 1.96	50.28 ± 3.24	48.98 ± 2.09
SI *	1.28	1.99	-	-
DOX-Dex-pAEMA	44.99 ± 2.95	45.16 ± 2.72	44.97 ± 1.69	37.38 ± 4.32
SI	0.99	0.83	-	-
DOX-SL-Dex-*g*-pAEMA	26.67 ± 1.65	22.65 ± 2.56	63.91 ± 3.36	59.71 ± 3.07
SI	2.40	2.64	-	-
Dex-g-pAEMA	>500	>500	>500	>500
SI	1.00	1.00	-	-

* SI is defined as the IC50 values of normal cells/IC50 values of cancer cells.

## Data Availability

The original contributions presented in this study are included in the article. Further inquiries can be directed to the corresponding author.
